# Supplementary material for: Glioma-derived CCL2 and CCL7 mediate migration of immune suppressive CCR2+/CX3CR1+ M-MDSCs into the tumor microenvironment in a redundant manner
Source: Front Immunol. 2023 Jan 4;13:993444. doi: 10.3389/fimmu.2022.993444 (PMC9854274; doi:10.3389/fimmu.2022.993444)
Supplement: Supplementary file 1 [file DataSheet_1.pdf]

Supplementary Material

Supplementary Figures

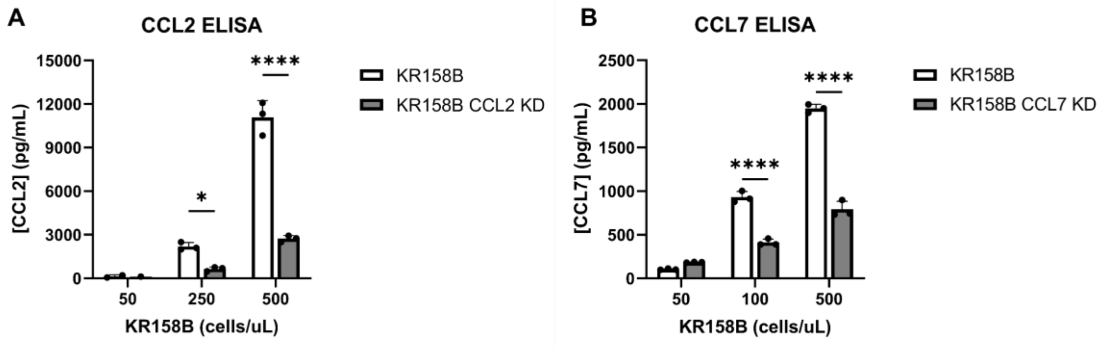

Supplementary Figure 1. KR158B glioma cells express CCR2 ligands CCL2 and CCL7. (A-B) ELISA results confirming the production of **(A)** CCL2 and **(B)** CCL7 respectively from the KR158B and respective KR158B-chemokine knockdown glioma cell lines. Two-way ANOVA statistics was conducted (Dunnett's multiple comparisons test). p-values: 0.0332(*), 0.0021(**), 0.0002(***), <0.0001(****)

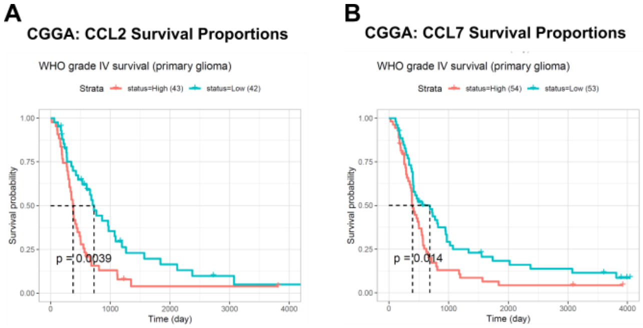

Supplementary Figure 2. High CCL2 and CCL7 expression is associated with negative prognosis for patients with glioblastoma in the CGGA dataset. Chinese Glioma Genome Atlas survival analysis. Kaplan-Meyer survival curves of WHO grade IV GBM patients, mRNA expression of **(A)** CCL2 **(B)** CCL7. Patients were stratified in high and low populations using the median gene expression value. Log-rank (Mantel-Cox) test was conducted on high vs low expressing cohorts.

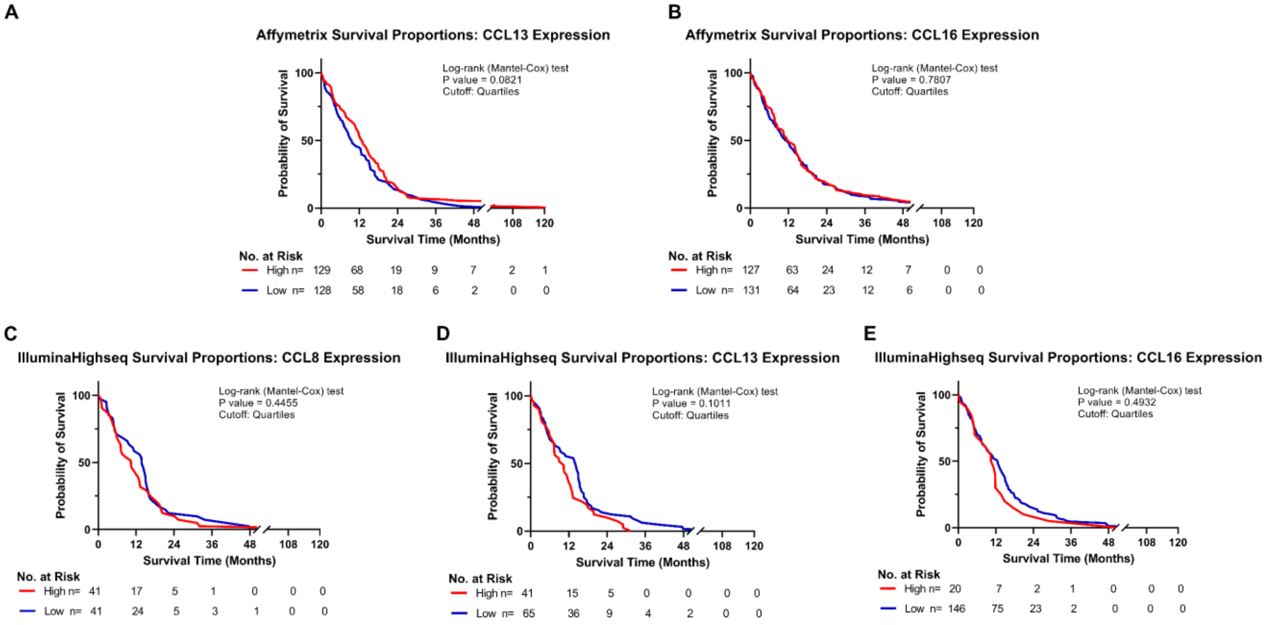

Supplementary Figure 3. Additional human CCR2 chemokine ligands do not confer negative prognosis for patients with glioblastoma. (A-B) Kaplan-Meyer survival curves of GBM patients based on Affymetrix gene expression profiles of **(A)** CCL13 **(B)** CCL16 **(C-E)** Kaplan-Meyer survival curves of GBM patients based on Illumina Highseq expression profiles of **(C)** CCL8 **(D)** CCL13 **(E)** CCL16 mined from TCGA database. High and low cohorts are stratified as top and bottom quartiles, respectively. Number at risk indicates surviving patients in each cohort at the respective timepoints of analysis. Log-rank (Mantel-Cox) test was conducted on high vs low expressing cohorts.

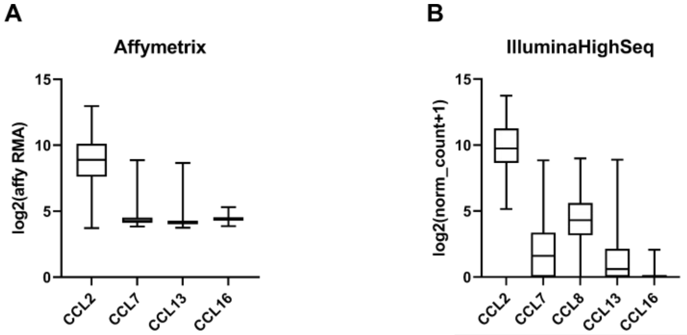

	CCL2	CCL7	CCL13	CCL16
Number of values	518	518	518	518
Minimum	3.722	3.842	3.760	3.869
25% Percentile	7.622	4.116	4.014	4.293
Median	8.893	4.259	4.151	4.420
75% Percentile	10.12	4.523	4.289	4.545
Maximum	12.97	8.865	8.654	5.310
Range	9.248	5.023	4.894	1.441

	CCL2	CCL7	CCL8	CCL13	CCL16
Number of values	166	166	166	166	166
Minimum	5.154	0.000	0.000	0.000	0.000
25% Percentile	8.646	0.000	3.172	0.000	0.000
Median	9.745	1.602	4.309	0.6093	0.000
75% Percentile	11.27	3.371	5.615	2.138	0.000
Maximum	13.74	8.844	8.993	8.898	2.069
Range	8.584	8.844	8.993	8.898	2.069

Supplementary Figure 4. Descriptive statistics of TCGA GBM survival analysis for CCR2 chemokine ligands CCL2, CCL7, CCL8, CCL13, and CCL16. **(A)** Affymetrix gene expression cohort **(B)** Illumina Highseq gene expression cohort. Number of patients analyzed, median, range, and quartiles are displayed. Quartiles were used to stratify high and low populations in survival analysis.

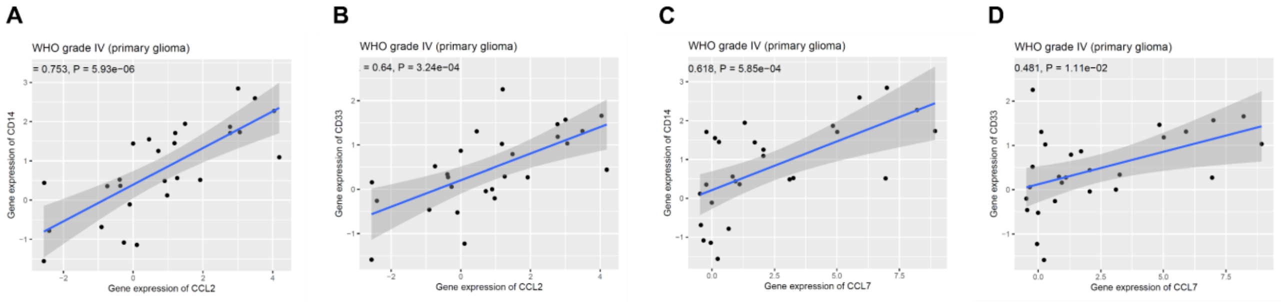

Supplementary Figure 5. CCL2 and CCL7 are positively correlated with M-MDSC markers in human GBM. Correlations of CCL2, CCL7 and M-MDSC markers from the CGGA database. WHO grade IV patients were selected for gene expression correlations **(A)** CD14 vs CCL2 **(B)** CD33 vs CCL2 **(C)** CD14 vs CCL7 **(D)** CD33 vs CCL7. Sample correlation coefficient and p-value are displayed.

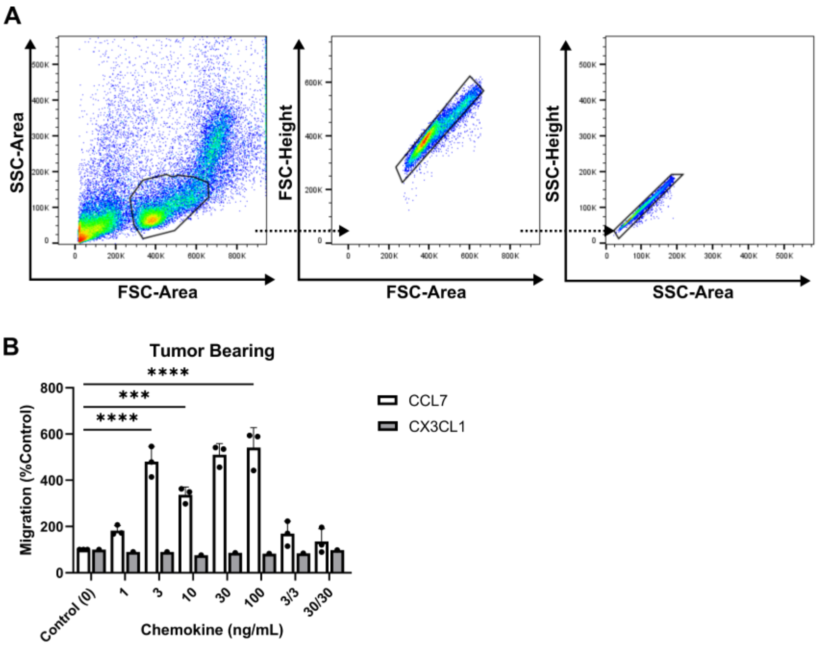

Supplementary Figure 6. CCR2$^+$/CX3CR1$^+$ cells do not migrate to soluble CX3CL1. (A) Representative flow cytometry gating strategy for Figure 4A in vitro migration experiments. Identical gating strategy was applied to all other conditions in that figure. **(B)** Migration to recombinant CCL7 (n=3) and recombinant soluble CX3CL1(n=1) of CCR2/CX3CR1 expressing cells derived from glioma bearing animals. Graph depicts no migration to recombinant CX3CL1. Two-way ANOVA statistics was conducted (Dunnett's multiple comparisons test). Differences are compared to the control (0) condition. p-values: 0.0332(*), 0.0021(**), 0.0002(***), <0.0001(****)

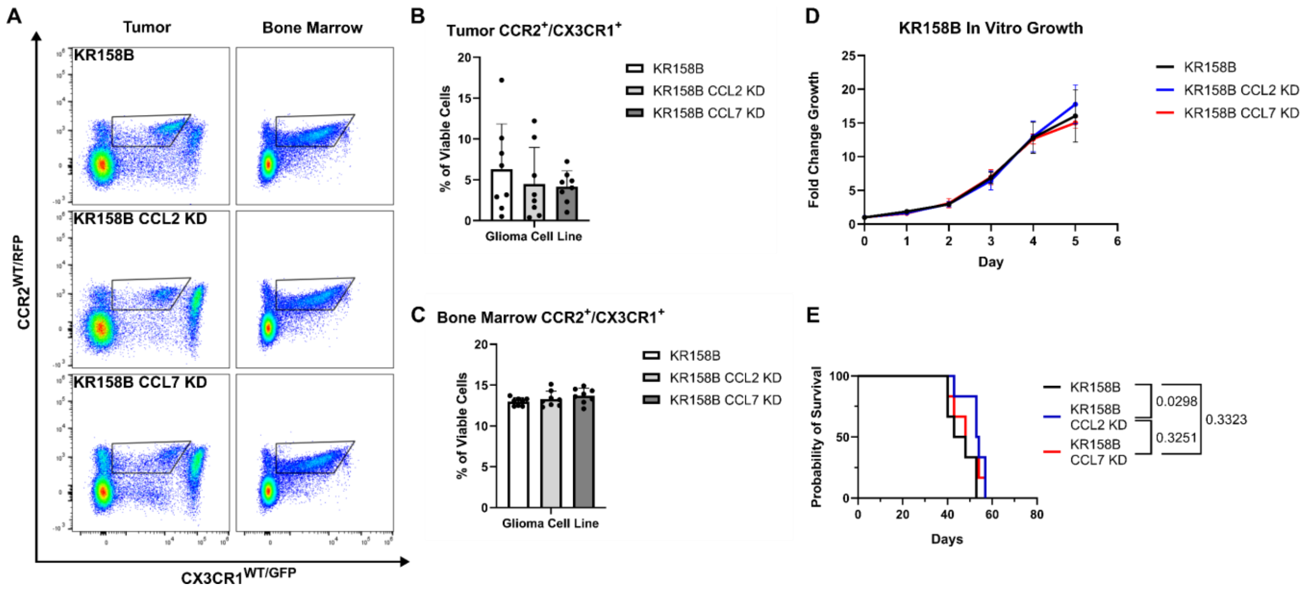

Supplementary Figure 7. Infiltration of CCR2⁺/CX3CR1⁺ cells into tumors is equivalent in mice implanted with KR158B, KR158B CCL2 KD, or KR158B CCL7 KD tumors. A) Representative flow plots depicting equivalent populations of CCR2/CX3CR1-expressing cells in the chemokine knockdown tumors compared to the KR158B tumor. No significant change in this population was observed in the bone marrow. **(B-C)** Graphs depicting the percent of CCR2/CX3CR1-expressing cells in the tumors and bone marrow of mice implanted with KR158B, KR158B CCL2 KD or KR158B CCL7 KD cell lines (n=8). **(D)** In vitro growth analysis of KR158B, KR158B CCL2 KD, and KR158B CCL7 KD glioma cell lines. CyQUANT Cell Proliferation dye was used to assess growth. Data is displayed as fold change growth from day 0 to day 5. **(E)** Survival analysis of KR158B, KR158B CCL2 KD, and KR158B CCL7 KD gliomas implanted in wildtype C57BL/6 mice. Two-way ANOVA statistical analysis was conducted for infiltrating cell populations (Dunnett's multiple comparisons test). p-values: 0.0332(*), 0.0021(**), 0.0002(***), <0.0001(****) Log-rank (Mantel-Cox) test was conducted on survival of KR158B, KR158B CCL2 KD, and KR158B CCL7 KD.

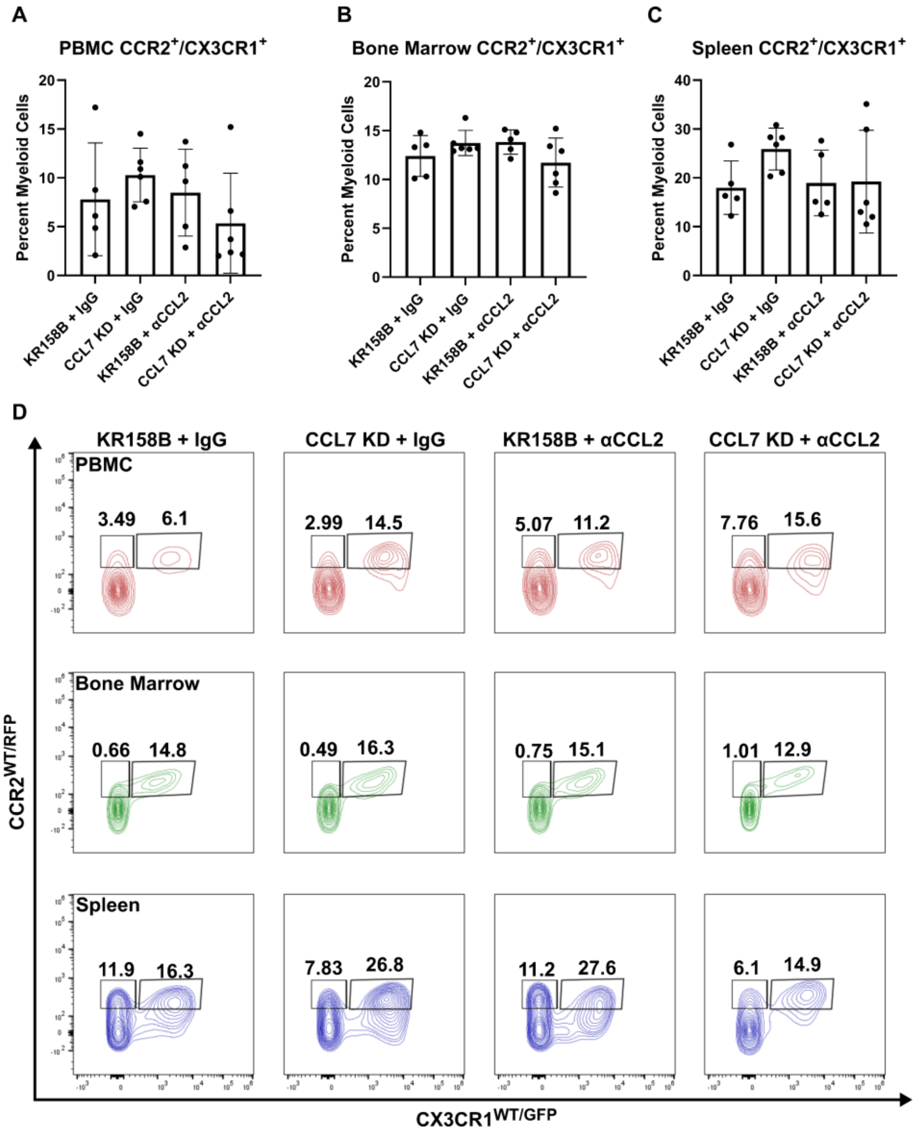

Supplementary Figure 8. In vivo targeting of CCL2 and CCL7 does not impact CCR2 and CX3CR1 populations in peripheral tissues. (A-C) Graphs depicting percentage of live, CD45^{+}, CD11b^{+}, CCR2^{+}/ CX3CR1^{+} cells in non-tumor peripheral compartments**. (D)** Representative flow cytometry plots showing no statistically significant changes in CCR2^{+}/ CX3CR1^{+} cells derived from PBMC, bone marrow, and spleen. Displayed plots are gated on live, CD45, CD11b positive cells. Example gating strategy can be found in Supplementary Figure 11A.

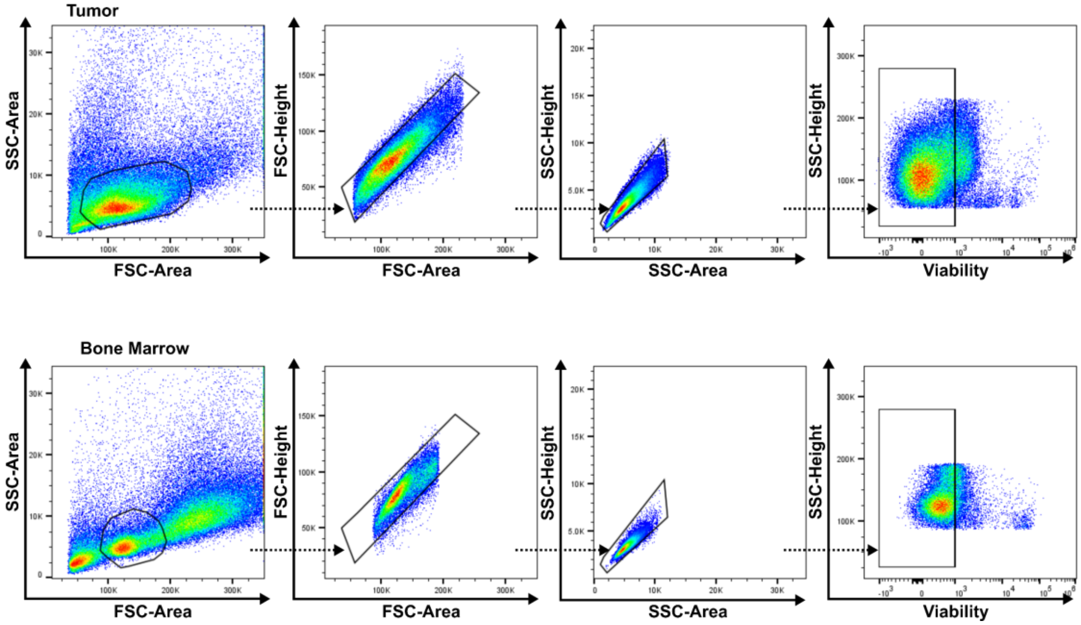

Supplementary Figure 9. Representative flow cytometry gating strategy for Supplementary Figure 7A in vivo migration of CCR2^{+}/CX3CR1^{+} cells in KR158B, KR158B CCL2 KD, and KR158B CCL7 KD: tumor (top) and bone marrow (bottom). Identical gating strategy was applied to all other conditions in that panel.

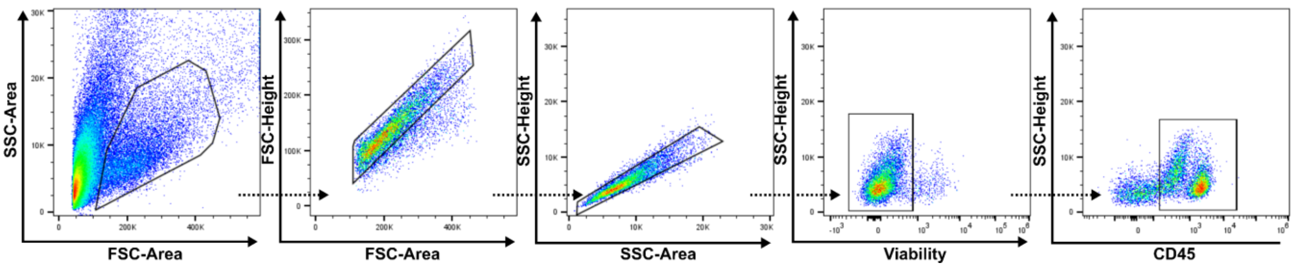

Supplementary Figure 10. Representative flow cytometry gating strategy for Figure 1B analysis of tumor infiltrating CCR2^{+}/CX3CR1^{+} cells in control and chimeric mice. Identical gating strategy was applied to all other conditions.

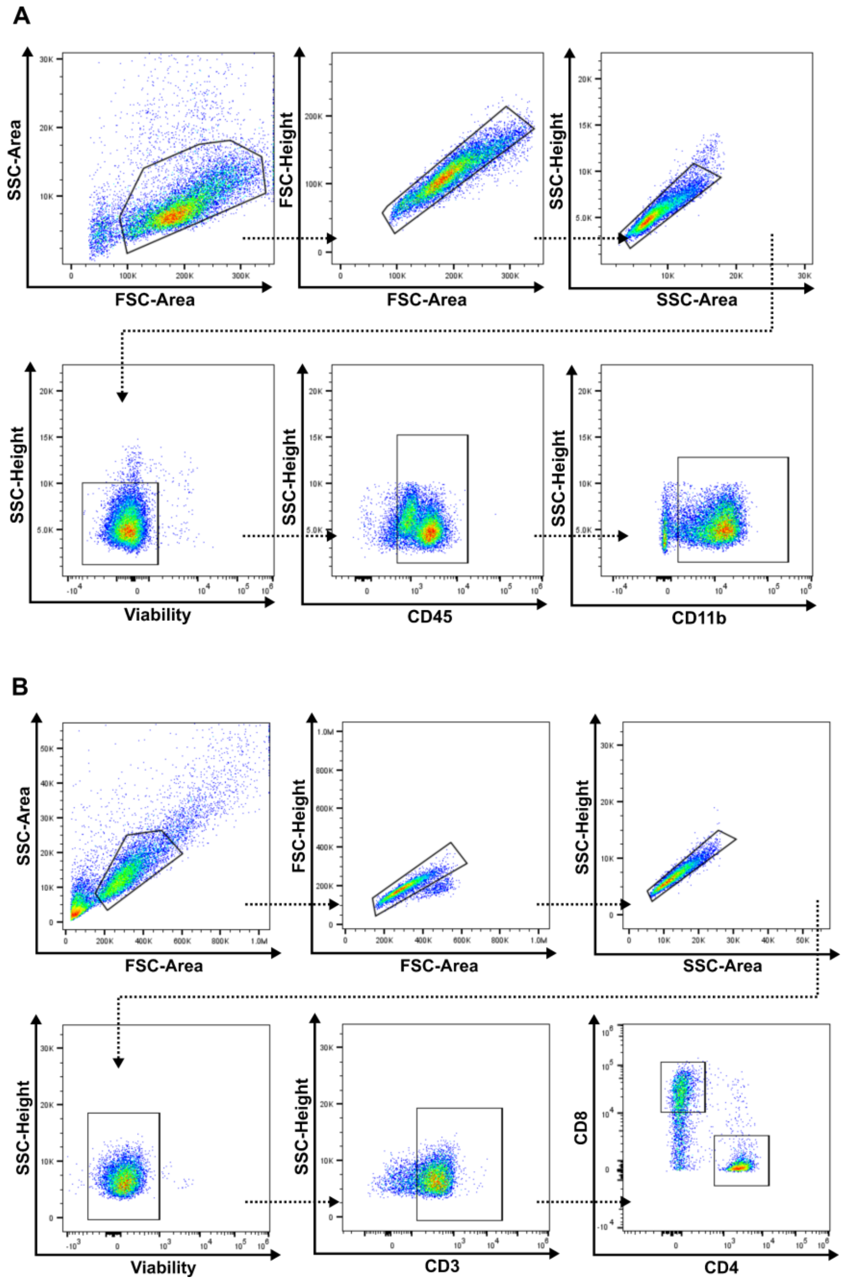

Supplementary Figure 11. (A) Representative flow cytometry gating strategy for Figure 2A post magnetically activated cell sorting. Identical gating strategy was applied to all other conditions in that panel. **(B)** Representative flow cytometry gating strategy for Figure 2B T cell suppression assay. CD4 and CD8 T cell proliferation were analyzed simultaneously. Identical gating strategy was applied to all other conditions in that panel.

Supplementary Table 1

Flow Cytometry

Protein Marker	Fluorophore	Company	Catalog #	Dilution Factor
CellTrace	FarRed	Invitrogen	C34564	1ul/1 x 10^6 cells
CD11b	BV421	Biolegend	101251	1:100
CD3	BV510	Biolegend	100234	1:100
CD4	FITC	Biolegend	100510	1:100
CD45	Alexa700	Biolegend	103128	1:200
CD8	PE	Biolegend	100708	1:100
LY6G	PerCP	Biolegend	127654	1:100
LY6C	BV785	Biolegend	128041	1:100
Viability	Pacific Blue	Invitrogen	L34963	1µl/mL

Biolegend, San Diego CA; Invitrogen, Carlsbad, CA
